# Safe surgical technique: reconstruction of the sternoclavicular joint for posttraumatic arthritis after posterior sternoclavicular dislocation

**DOI:** 10.1186/1754-9493-7-38

**Published:** 2013-12-31

**Authors:** Philip F Stahel, Brian Barlow, Frances Tepolt, Katharine Mangan, Cyril Mauffrey

**Affiliations:** 1 Department of Orthopaedics, Denver Health Medical Center, University of Colorado, School of Medicine, Denver, CO 80204, USA; 2 Department of Orthopaedics, Naval Medical Center, San Diego, CA 92134, USA; 3 Department of Orthopaedics, Denver Health Medical Center, University of Colorado, School of Medicine, 777 Bannock Street, Denver, CO 80204, USA

## Abstract

Posttraumatic sternoclavicular arthritis related to chronic ligamentous instability after posterior sternoclavicular dislocation represents a rare but challenging problem. The current article in the *Journal’s* “Safe Surgical Technique” series describes a successful salvage procedure by partial resection of the medial clavicle and ligamentous reconstruction of the sternoclavicular joint with a figure-of-eight semitendinosus allograft interposition arthroplasty.

## Background

Since the first description of a traumatic posterior sternoclavicular dislocation 170 years ago [1], several anecdotal case reports have been published on this rare injury pattern [2-8]. While uncommon, posterior sternoclavicular dislocations represent potentially life-threatening injuries due to the close proximity of the posterior mediastinal structures [9,10]. Aside from the risk of a vascular injury [11], tracheal tears and esophageal compression have been described, which lead to acute dyspnea and dysphagia [12]. Additional associated injuries include a traumatic vocal cord palsy [13] and brachial plexus injury [14]. Establishing an early diagnosis is difficult since these injuries are frequently missed [5,15,16]. Clinically, patients with traumatic posterior sternoclavicular dislocations are in significant pain, potentially associated with venous congestion, shortness of breath, and dysphagia. The medial end of the clavicle is typically palpable lateral to the jugular notch, and when displaced posteriorly, the corner of the sternum is exposed to palpation. As the clinical examination frequently remains equivocal, radiographic studies are required to establish the diagnosis [5,16,17]. Conventional radiographs are not sensitive for posterior sternoclavicular dislocations, and computed tomography (CT) represents the imaging modality of choice [18,19].

### Acute management strategies

Due to the proximity of the sternoclavicular joint to vulnerable structures in the superior mediastinum, dislocations must be reduced as early as possible [20]. Forty to fifty percent of all posterior sternoclavicular joint dislocations are successfully managed by closed reduction [21,22]. The most frequently described reduction maneuver consists of an ‘abduction/traction’-technique with the patient placed in supine position with a bump or sandbag between the shoulders, and gradual traction applied to the abducted arm, with slow progression to extension [23]. If the reduction maneuver is successful, the clavicle reduces with an audible ‘popping’ sound. Some authors recommend the use of a percutaneous sterile towel clip to grasp the medial clavicle with lateral and anterior traction [23]. About 50% of all closed reduction attempts are unsuccessful and place the patient at risk of additional harm [24]. Severe complications have been reported after closed reduction maneuvers. As an example, a “near miss” complication has been described in which the medial clavicle perforated the right pulmonary artery, and surgical exploration revealed that acute bleeding was prevented by the clavicle compressing the artery [25]. In this circumstance, a closed reduction maneuver would have likely resulted in unforeseen disaster. Thus, multiple authors recommend the early open surgical treatment of posterior sternoclavicular dislocations [26-29]. The ‘classic’ operative technique described by Burrows in 1951 consists of a subclavius tenodesis for stabilization of the sternoclavicular joint [30]. Multiple additional surgical techniques have more recently been described, including fixation with cannulated screws [28], bridge plating [31,32], cable fixation [33], artificial ligament reconstruction [34], and tendon reconstruction of the disrupted capsular/ligamentous complex [35,36]. Of note, the use of Kirschner wires has been abandoned due to the risk of pin migration resulting in delayed penetration of vascular structures [37,38]. Interpositional arthroplasty utilizing the sternal head of the sternocleidomastoid muscle has been recommended in conjunction with resection of the medial clavicle [39]. Resection of the medial clavicle alone, however, has been associated with poor outcomes, particularly in cases with residual ligamentous instability [40-42].

### Applied surgical anatomy

**Figure 1:**
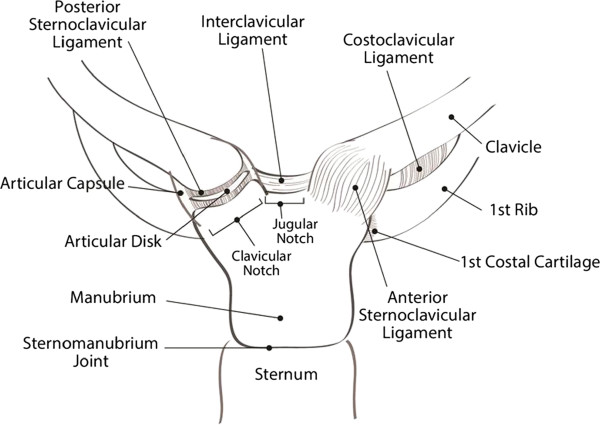
Ligamentous structures around the sternoclavicular joint.

The sternoclavicular joint represents the only ‘true’ articulation between the shoulder and the axial skeleton. The articular surface of the medial clavicle is much larger than the sternal joint surface, leaving the sternoclavicular joint with less than 50% congruity between its bony components. The intraarticular disk ligament connects the synchondral junction of the first rib with the manubrium and divides the sternoclavicular joint into two separate joint spaces. Joint stability relies nearly completely on the integrity of the joint capsule and associated ligamentous complex, including the anterior and posterior sternoclavicular ligament and the costoclavicular (“rhomboid”) ligament (Figure 1). Posterior dislocations typically result in disruption of the anterior sternoclavicular ligament, strain or disruption of the costoclavicular ligament, and preservation of the posterior sternoclavicular ligament due to the antero-posterior vector of the impacting force. Traumatic disruption of the anterior joint capsule leads to the distal end of the clavicle inadequately resisting against axial loading forces, and hinging of the medial end of the clavicle over the first rib. The resulting ligamentous instability represents a major root cause for early posttraumatic sternoclavicular arthritis after traumatic joint dislocations.

**Figure 2:**
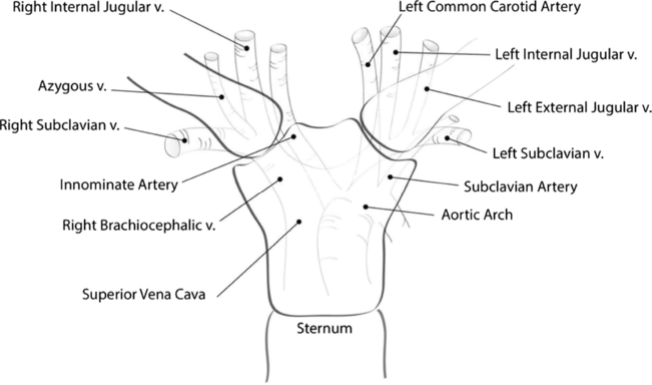
Applied surgical anatomy to the vascular structures posterior to the sternoclavicular joint.

A detailed understanding of the anatomy of multiple vital structures posterior to the sternoclavicular joint is of crucial importance for a safe and coherent decision-making process in the management of traumatic posterior sternoclavicular dislocations. The posterior vascular structures ‘at risk’ for traumatic lacerations and iatrogenic intraoperative injuries are depicted in Figure 2. These include the innominate vein, left subclavian vein, internal and external jugular veins, and left common carotid artery, for *left*-sided dislocations, and the innominate vein, right internal and external jugular veins, and innominate artery, for *right*-sided dislocations. Multiple muscles behind the sternoclavicular joint (scaleni, sternohyoid, sternothyroid) act as a protective ‘buffer’ anterior to these vascular structures. The vagus nerve, phrenic nerve, trachea and esophagus (not shown in the figure) are also at significant risk of traumatic injury from posterior sternoclavicular dislocations. Furthermore, the apical parts of the lungs are at risk of traumatic or iatrogenic injury, which may result in a pneumothorax. The surgeon responsible for managing an acute or chronic dislocation of the sternoclavicular joint must understand the close proximity of these vulnerable structures in the upper mediastinum.

## Case presentation

**Figure 3:**
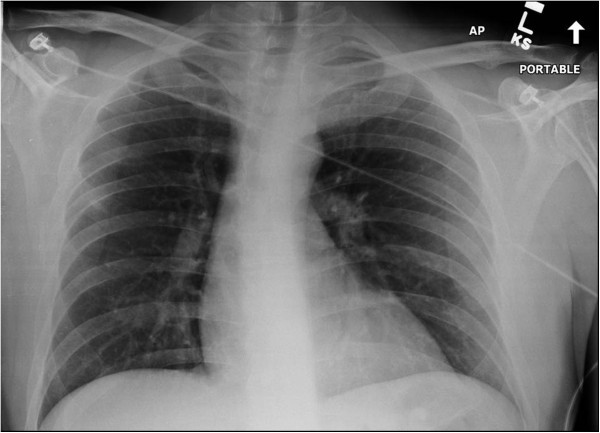
**Chest radiograph from a 45-year old patient who sustained a left posterior sternoclavicular joint dislocation after a fall on his left shoulder in a skiing accident.** Note that the subtle signs of the incongruent anatomy of the left sternoclavicular joint, which is frequently missed on plain radiographs.

**Figure 4:**
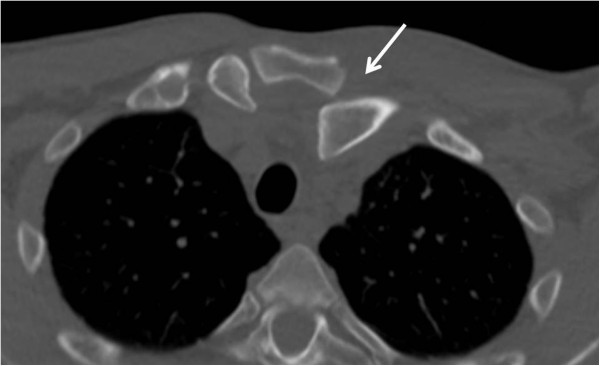
The axial CT scan confirms the clinical suspicion of a left posterior sternoclavicular joint dislocation (arrow).

**Figure 5:**
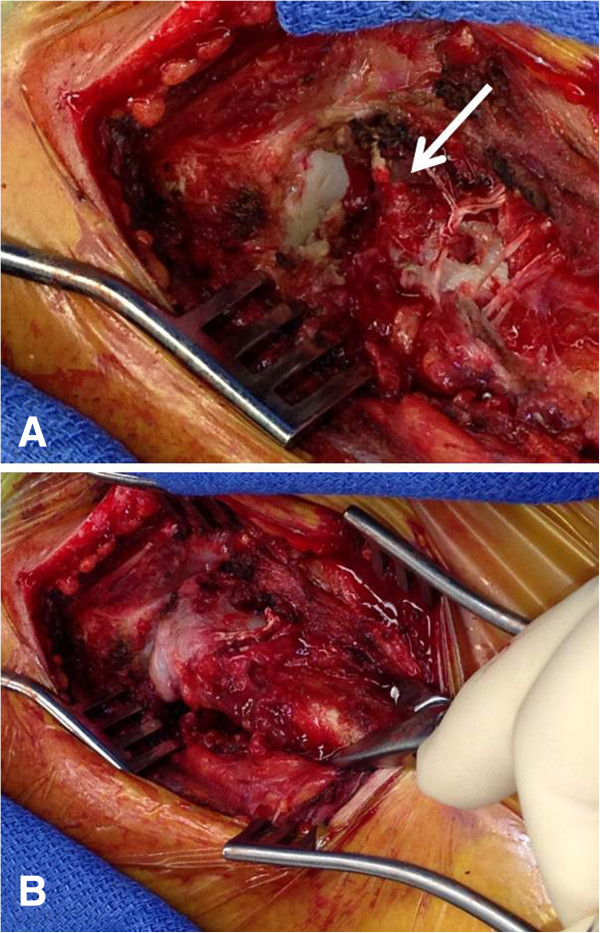
**Intraoperative demonstration of the traumatic posterior sternoclavicular dislocation.** Panel **A** shows complete traumatic disruption of the anterior joint capsule and anterior sternoclavicular ligament (arrow) with the sternal joint exposed and the medial clavicle locked behind the manubrium **(A)**. Open reduction was achieved by the use of a serrated bone clamp **(B)**.

**Figure 6:**
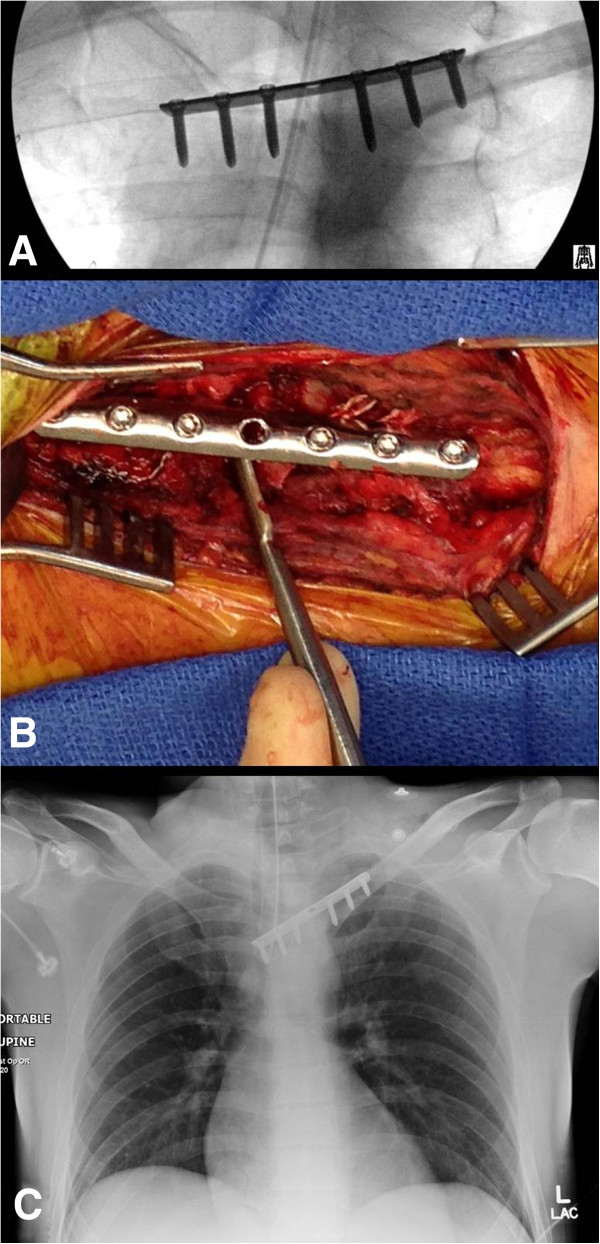
**Bridge plating of the left sternoclavicular joint using a 3.5 mm third-tubular locking plate.** Panel **A** shows the intraoperative fluoroscopic view after bridge plate fixation. The periosteal elevator in panel **B** denotes the sternoclavicular joint space below the plate. An on-table chest radiograph was obtained to rule out an iatrogenic left-side pneumothorax **(C)**.

A 45 year-old right hand dominant gentleman sustained a fall on his left shoulder in a skiing accident in Colorado. He was in significant pain and shortness of breath and therefore referred to a local hospital for evaluation. A chest radiograph was unremarkable and particularly revealed no evidence of a pneumothorax, hemothorax, or widened mediastinum (Figure 3). Due to significant tenderness over the left sternoclavicular joint, a CT scan was obtained which demonstrated a locked posterior sternoclavicular dislocation (Figure 4). The patient was transferred to our academic Level 1 trauma center for definitive care. On arrival in our emergency department, the patient complained of significant pain in his chest and left shoulder, as well as left-sided neck pain. The upper airway was patent, and the patient was hemodynamically stable and fully awake, alert, and oriented, with a Glasgow Coma Scale (GCS) score of 15. A CT-angiogram of the neck revealed no sign of an acute cervical spine injury or associated vascular injury (not shown). Due to the locked position of the clavicle behind the manubrium (Figure 4), no attempt for a closed reduction maneuver was made, and the patient was consented for surgical open reduction and internal fixation. As part of the pre-operative planning, we ensured immediate availability of a vascular surgeon, and the presence of a sternal saw and vascular set in the operating room, for immediate access in case of an accidental intraoperative vascular laceration. The patient was placed in supine position with a bump placed under the left shoulder. The left upper extremity freely draped. The surgical approach consisted of an incision of 10 cm length along the superior border of the medial clavicle, with a slight curve just medial to the sternoclavicular joint, across the midline of the manubrium. A full-thickness soft tissue dissection was performed down to the left sternoclavicular joint utilizing a meticulous dissection technique to avoid accidental transsection of the anterior capsular ligament and the costoclavicular ligament. The head of the medial clavicle was found to be incarcerated behind the manubrium and irreducible due to the interposition of the torn joint capsule and ruptured anterior sternoclavicular ligament (arrow in Figure 5A). The interposing joint capsule was therefore partially removed and the sternoclavicular joint was exposed and reduced with anterior and lateral traction using a serrated reduction clamp (Figure 5B). The joint was found to be grossly unstable after reduction due to incompetence of the anterior capsule and anterior sternoclavicular ligament. The posterior joint capsule and the costoclavicular ligament were preserved. The surgical plan consisted of anatomic joint reduction and bridge plate fixation using a 3.5 mm one-third-tubular locking plate (Synthes, Paoli, PA). The plate was placed on the anterior/superior border of the medial clavicle and spanned across the midline of the manubrium (Figure 6A). Unicortical locking head screw fixation was chosen to minimize the risk of unintentional injury associated with drilling across the far cortex. Anatomic reduction was achieved and maintained with the bridging locking plate fixation (Figure 6B). The wound was washed out and closed in layers. An on-table chest radiograph was obtained to rule out an iatrogenic left-side pneumothorax (Figure 6C). The patient tolerated the procedure well and reported minimal postoperative pain. His left shoulder was immobilized in a sling for pain control, followed by gradual mobilization with pendulum exercises.

**Figure 7:**
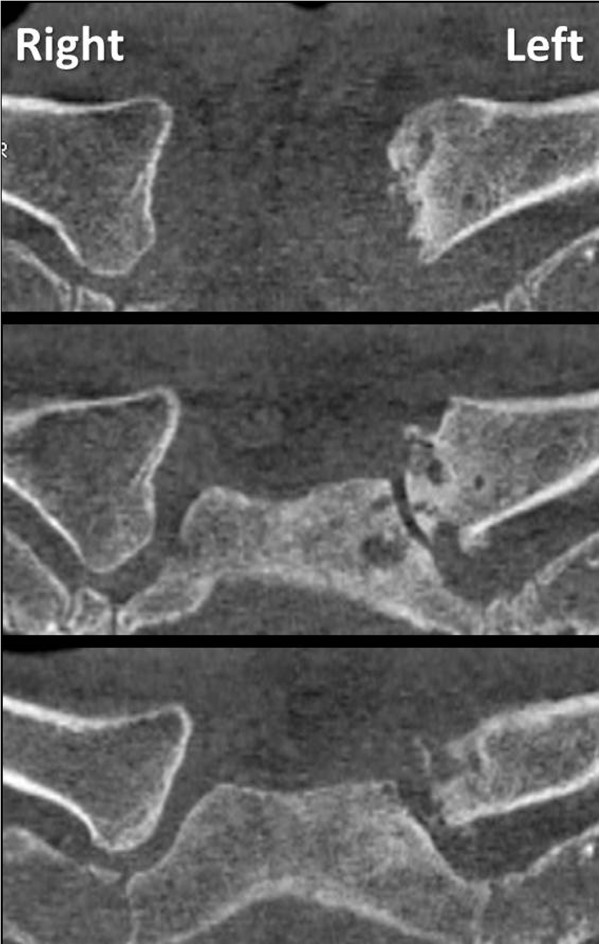
Follow-up CT scan at 9 months post injury (7 months after hardware removal) demonstrates early posttraumatic arthritis of the left sternoclavicular joint, compared to the contralateral (uninjured) right side.

The patient was discharged from the hospital on postoperative day 1. He appeared to have an excellent subjective outcome with full and painless range of motion in the left shoulder. At 2 months post injury, he underwent an early hardware removal of the locking plate by a local orthopedic surgeon. Initially, the patient continued to have an uneventful recovery, unrestricted in his daily activities. Within 6 months, however, he started developing progressive, ultimately excruciating pain over the left sternoclavicular joint, dramatically restricting his daily functional activities. The symptoms deteriorated to a point at which the patient awakened from sleep every hour secondary to pain. The symptoms were refractory to the chronic intake of non-steroidal inflammatory agents (ibuprofen). A follow-up CT scan at 9 months post injury revealed significant posttraumatic arthritis of the left sternoclavicular joint, in conjunction with recurrent posterior subluxation of the medial clavicle (Figure 7). The patient presented again to our institution and an indication was placed for surgical revision due to the symptomatic instability and progressive posttraumatic arthritis, which likely occurred secondary to early hardware removal of the bridging locking plate. The surgical plan entailed a partial resection arthroplasty of the medial clavicle in conjunction with a ligamentous reconstruction with a semitendinosus allograft tendon woven in a figure-of-eight pattern through drill holes in the manubrium and residual medial clavicle. This particular technique has been shown to improve stability compared to local tissue transfers, e.g. using the ‘classic’ Burrows technique [30], and has been associated with excellent subjective outcomes in cases of chronic pain and posttraumatic joint instability [43].

### Surgical technique

**Figure 8:**
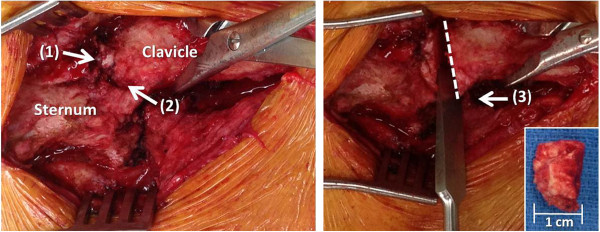
**Surgical revision reveals posttraumatic arthritis at the medial clavicular head (1) and an incompetent anterior sternoclavicular ligament (2).** A partial resection arthroplasty of the medial clavicle was performed, with the osteotomy level just medial to the insertion of the costoclavicular ligament (3). The resected medial clavicle head (inserted panel) should be less than 1 cm wide in order to avoid jeopardizing the integrity of the costoclavicular ligament.

The scar from the previous surgical approach to the left sternoclavicular joint was used for the revision procedure, as described above. A full-thickness dissection was performed through scar tissue down to the sternoclavicular joint. The ligamentous instability was verified intraoperatively by stress testing with a serrated reduction clamp (Figure 8). Progressive arthritic changes were found at the medial clavicular joint, with multiple osteophytes and exposed bone related to grade IV degenerative cartilage lesions (arrow 1 in Figure 8). In addition, the anterior sternoclavicular ligament and anterior joint capsule were incompetent (arrow 2 in Figure 8), and the medial clavicle was subluxated posteriorly. The medial clavicle was resected by creating multiple parallel 2.5 mm drillholes in antero-posterior direction which were connected with an osteotome to finalize the osteotomy (Figure 8).

#### Technical trick

*Meticulous care has to be taken not to plunge across the far cortex of the medial clavicle, due to the close proximity of posterior vascular structures, and not to extend the resection more than 1 cm laterally in order to preserve the insertion of the costoclavicular ligament. A malleable retractor is placed posterior to the clavicle to protect from the tip of the drill bit, and the osteotomy across the far cortex is carefully completed by the use of an osteotome. The resected part of the medial clavicle should not extend beyond 1 cm in length* (small insert in Figure 8).

**Figure 9:**
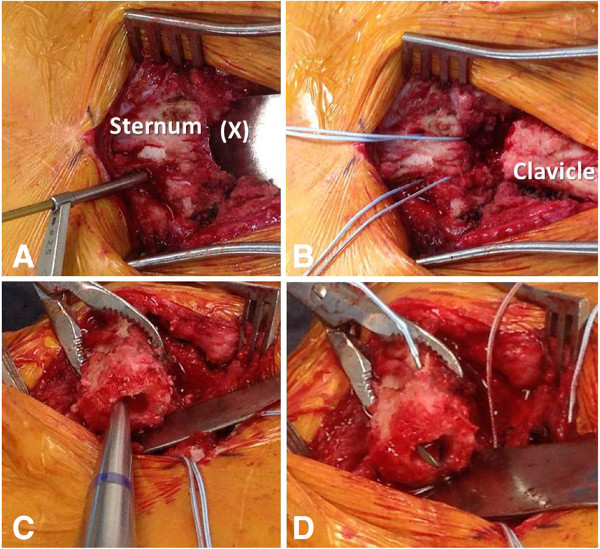
**Surgical technique for ligamentous reconstruction of the sternoclavicular joint.** The marking (X) in panel **A** denotes a protective malleable retractor to protect from accidental perforation of posterior mediastinal structures. Panel **B** demonstrates the placement of Fiberwire sutures through the sternal drill holes. The medullary cavity of the clavicular end is cleared with a curette **(C)** and the needle of the Fiberwire suture is passed in an intramedullary fashion through the clavicular drill holes **(D)**.

**Figure 10:**
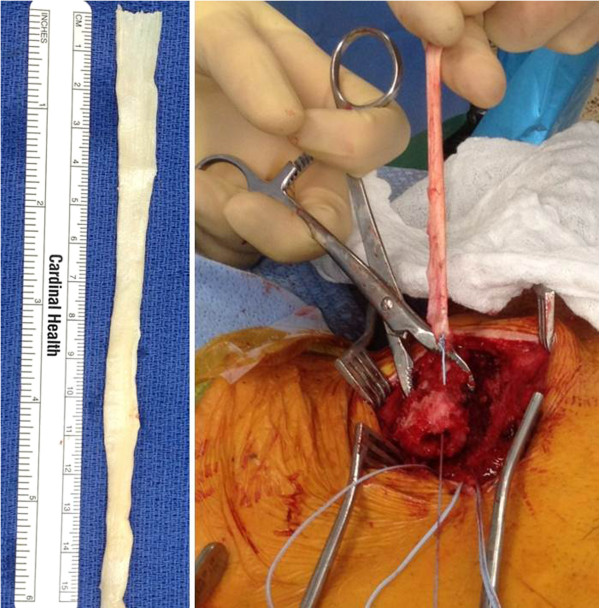
**Semitendinosus allograft tendon for ligamentous reconstruction of the sternoclavicular joint.** See text for details and explanations.

**Figure 11:**
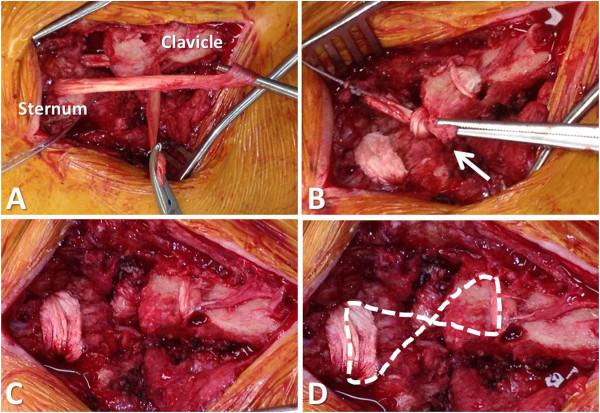
**Intraarticular interposition arthroplasty by figure-of-eight semitendinosus allograft reconstruction.** The tendon is passed through the four drill-holes with the use of the temporary sutures and tensioned in anatomic position **(A)**. A double knot is then tied with the ends of the semitendinosus graft **(B)**. Panel **C** demonstrates the final reconstruction. The dotted line in panel **D** outlines the figure-of-eight position of the tendon graft.

In the present case, the costoclavicular ligament was intact and competent (arrow 3 in Figure 8). The surgical plan was therefore restricted to the exclusive reconstruction of the anterior sternoclavicular ligament. We applied a technique of intraarticular ligament transfer with a semitendinosus allograft ‘figure-of-eight’ reconstruction (Figures 9, 10, 11). Two drillholes each are placed at the level of the manubrium and the residual medial clavicle in antero-posterior direction. Subperiosteal dissection is performed around the lateral end of the manubrium and medial end of the residual clavicle to allow placement of a malleable retractor (Figure 9A) for protection of the posterior cortex from the tip of the drill bit.

#### Technical trick

The antero-posterior drill holes are placed at a distance of 1 cm medial to the lateral sternal border, and 1 cm lateral to the site of the medial clavicular resection, to ensure no breach of the residual cortical bridge. We recommend using consecutive drill bit sizes in ascending order of 2.5 mm, 3.5 mm, and 4.5 mm to ensure adequacy of placement of the intraarticular drill holes for the ligament transfer. A drill hole of 4.5 mm diameter is the minimum requirement for successful passing of the semitendinosus tendon graft.

Temporary sutures are passed through the sternum to mark the drill holes and facilitate passage of the tendon graft (Figure 9B). The medullary canal of the clavicle is then drilled with a 4.5 mm drill bit and opened with a curette (Figure 9C). A temporary suture is passed through the clavicular drill holes, exiting the medullary canal (Figure 9D). The semitendinosus allograft is secured on a braided non-absorbable suture (e.g. Fiberwire®, Arthrex, Naples, FL) and passed through the medullary canal of the clavicle (Figure 10). The tendon graft is then passed in a figure-of-eight position through the tagged drill holes in the clavicle and sternum (Figure 11).

#### Technical trick

*It is important to hold the sternoclavicular joint reduced in anatomic position prior to tying two consecutive knots with the ends of the tendon within the residual joint space. The knot of the tendon within the joint space serves as a ‘spacer’ for the resection arthroplasty of the medial clavicle* (Figure 11).

**Figure 12:**
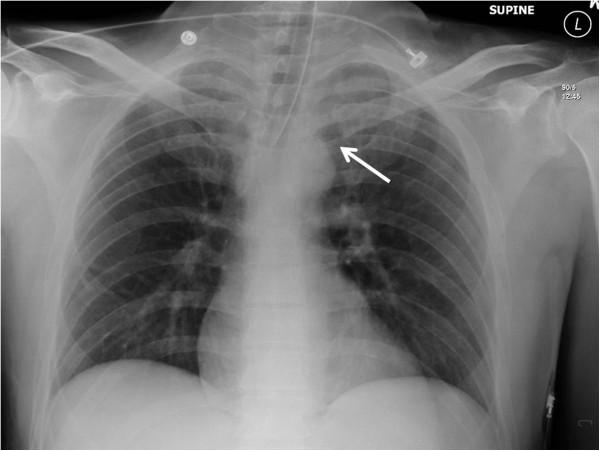
**On-table chest radiograph after the revision procedure was obtained to rule out an iatrogenic left-side pneumothorax.** The arrow denotes the resection arthroplasty of the left sternoclavicular joint.

A braided non-absorbable suture (e.g. Fiberwire®) is passed through the tendon knot in several layers to secure the fixation. It is important to sink the tied knot from the suture in the posterior soft tissues, in order to avoid interference of the suture knot with the thin skin envelope over the sternoclavicular joint. After meticulous bleeding control, the wound is washed out and closed in layers. We choose to obtain an on-table chest Xray (Figure 12) for postoperative documentation and exclusion of an iatrogenic pneumothorax due to the close proximity of the surgical dissection and the pleural space.

**Figure 13:**
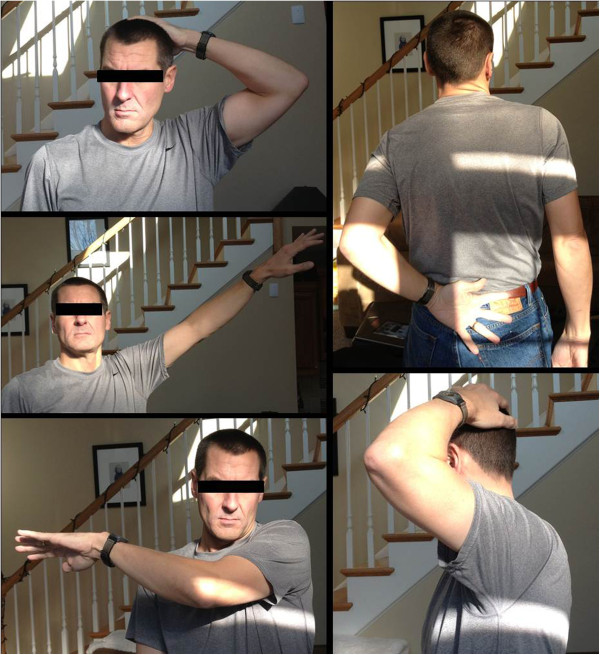
Functional outcome of the 45-year old patient described in this case report, at one month after the surgical revision of the left sternoclavicular joint.

The patient described in this case report recovered well and had an excellent and painless function of the left shoulder (Figure 13). He was able to resume full activity without restrictions within one month after the surgical revision procedure.

## Conclusions

✓ Posterior sternoclavicular dislocations are rare injuries which are frequently missed. Delayed diagnosis may relate to the fact that a plain chest radiograph is not sensitive in detecting this injury pattern (Figure 3). The ‘gold standard’ for establishing diagnosis is by clinical examination and CT scan (Figure 4).

✓ Many authors recommend the early open reduction and surgical fixation of posterior sternoclavicular dislocations due to limited proven success of closed reduction, and the potential for iatrogenic injuries associated with attempted closed reduction maneuvers [26-29]. The use of Kirschner wires for joint transfixation is strongly discouraged due to the risk of pin migration with penetration into the great vessels in the upper mediastinum [37,38].

✓ Locked plate fixation represents a feasible new treatment option for the acute management of traumatic posterior sternoclavicular dislocations, by restoring joint stability and allowing early functional rehabilitation [31]. The success of this technique is confirmed by the present case report, in which the patient had an excellent and uneventful postoperative recovery after locked plate fixation.

✓ Preemptive hardware removal after locked plate fixation, specifically within less than 3 months postoperatively, should be strongly discouraged due to the risk of recurrent joint instability as a root cause of early symptomatic posttraumatic arthritis. In analogy to the established plate fixation across joints in other anatomic locations (e.g. pubic symphysis plating), the consideration should be made of maintaining implants for lifetime in asymptomatic patients.

✓ Multiple reconstruction techniques have been described for restoring stability of the sternoclavicular joint in cases of posttraumatic arthritis related to chronic ligamentous instability. The resection of the medial clavicle alone is associated with poor long-term outcomes and therefore discouraged in cases with residual ligamentous instability [40-42].

✓ The surgical technique described herein consists of anterior ligamentous complex reconstruction by tendon tissue woven in a figure-of-eight intraarticular interposition technique. This is performed in conjunction with partial resection of the medial clavicular head proximal to the insertion of the costoclavicular ligament. This technique appears to represent the treatment modality of choice for addressing chronic joint instability in conjunction with posttraumatic arthritis, based on the results of the present case report and the current peer-reviewed literature [42,43].

## Consent

Written informed consent was obtained from the patient for publication of this article.

## Competing interest

The authors declare no other competing interests related to this manuscript.

## Authors’ contributions

PFS designed the technical instructional article and performed the surgical procedure presented in this case report. BB and FT provided a first draft of the manuscript. BB commissioned the graphic artwork shown in Figures 1 and 2. KM provided the intraoperative pictures shown in Figures 8–11. CM assisted with conception of the manuscript and final revisions. All authors read and approved the final version of this article.
